# Effect of Age on Survival Outcome in Operated and Non-Operated Patients with Colon Cancer: A Population-Based Study

**DOI:** 10.1371/journal.pone.0147383

**Published:** 2016-01-20

**Authors:** ZhongHua Jiang, XiaoHong Wang, XueMing Tan, ZhiNing Fan

**Affiliations:** 1 Department of Digestive Endoscopy and Medical Center for Digestive Diseases, The Second Affiliated Hospital of Nanjing Medical University, Nanjing, China; 2 Department of Gastroenterology, The NO.1 People’s Hospital of Yancheng, Yancheng, Jiangsu, China; 3 Department of Gastroenterology, The Second Affiliated Hospital of Xuzhou Medical College, Xuzhou, Jiangsu, China; 4 Department of Digestive Endoscopy, The First Affiliated Hospital of Nanjing Medical University, Nanjing, China; Instituto Nacional do Câncer, BRAZIL

## Abstract

**Objective:**

To know the effect of age on survival outcome in operated and non-operated patients with colon cancer.

**Methods:**

From the Surveillance, Epidemiology, and End Results database, we identified 123,356 patients with colon cancer who were diagnosed between 1996 and 2005, grouped them as older or younger than 40 years and analyzed their 5-year cancer-specific survival (CSS) data, along with some risk factors, using Kaplan–Meier methods and multivariable Cox regression models.

**Results:**

The younger group had significantly higher pathological grades (*P*<0.001), more mucinous and signet-ring histology (*P*<0.001), advanced AJCC stage (*P*<0.001), and were more likely to undergo surgery (*P*<0.001). For surgically treated patients, age did not significantly affect 5-year CSS (younger: 66.7%; older: 67.3%; *P* = 0.86). Further analysis showed that age was an independent prognostic factor in stage I–IV disease (stage I: *P* = 0.001; *P*<0.001 for stages II–IV, in both uni- and multivariate analyses), but not for patients with unknown disease stage (*P* = 0.52). For non-surgically treated patients, age significantly affected 5-year CSS (younger: 16.2%; older: 12.9%; *P*<0.001) in univariate analysis; and was an independent prognostic factor (*P*<0.001) in multivariate analysis.

**Conclusion:**

The CSS rate for younger CC patients was at least as high as for older patients, although they presented with higher proportions of unfavorable factors and more advanced disease.

## Introduction

Colon cancer (CC) is one of the most common malignancies; combined with rectal cancer, it is the second leading cause of cancer-associated mortality in the United States [1, 2]. Generally, CC is thought to be a malignancy that mainly occurs in patients older than 50 years of age [3]. Despite the 2012 Annual Report to the Nation on Cancer report of a steady decline in the incidence of colorectal cancer (CRC) in the United States [4], its incidence among younger patients is disproportionately increasing, with an annual incidence increase of 2.6% in rectal cancer vs 0.2% in colon cancer [5–7]. In younger patients, CC is more likely to be diagnosed in later stages, and to be mucinous or poorly differentiated tumors, including signet ring carcinoma [8–11]. However, mixed results have been reported with regard to prognoses, with some studies indicating poorer outcomes for younger patients [12–15], and others showing their survival rates to be at least as favorable as their older counterparts [16–19]. A complication in our understanding of young-onset CC is the fact that most published studies on young-onset colorectal cancer (CRC) analyzed both colon and rectal cancer together, were limited by relatively small sample sizes or single referral centers, and/or did not adjust for potential confounding factors. Moreover, most previous studies’ inclusion criteria were limited by surgical resection, but did not consider the prognostic significance of age on non-operated patients. To further refine a comprehensive analysis of colon cancer in younger patients at a national, population-based level, we used data from the Surveillance, Epidemiology and End Results (SEER) registries to (a) focus on the clinicopathological features in younger patients, and (b) analyze the role of age in colon cancer-specific survival (CCSS) while controlling for disease factors and treatment factors.

## Materials and Methods

### Patients

The SEER program collects data on the most recent cancer incidence, prevalence, mortality, and survival data from 17 population-based cancer registries that represent approximately 28% of the US population [20]. SEER data contain no identifiers and are publicly available for studies of cancer-based epidemiology, health policy, and survival analysis. The National Cancer Institute’s SEER*Stat software (Surveillance Research Program, National Cancer Institute SEER*Stat software, www.seer.cancer.gov/seerstat Version 8.1.2) was used to identify patients who were diagnosed with invasive CC (C18.0–19.9) between 1996 and 2005. Only patients aged between 15 and 80 years were included. Patients were excluded if they had in situ TMN staging, or had another primary tumor besides colon cancer. Histology types were limited to adenocarcinoma (8150/3, 8210/3, 8261/3, 8263/3), mucinous adenocarcinoma (8480/3), and signet-ring cell carcinoma (8490/3). Tumor-node-metastasis classifications were based on the criteria of the American Joint Committee on Cancer (AJCC) Cancer Staging Manual (7th edition, 2010), by which criteria patients were restaged. Age, sex, race, histological type, AJCC stage, and CCSS were analyzed. Because the SEER data contain no adjuvant chemotherapy information, adjuvant chemotherapy was not assessed. The primary endpoint of the study was CCSS, which was calculated as the time between diagnosis and CC-specific death. Deaths caused by CC were treated as events; living patients or deaths from other causes were censored data.

### Ethics Statement

This study was based on the free public SEER database, which is an authoritative source of information on cancer incidence and survival in the United States. All relevant data were obtained from SEER*Stat (http://seer.cancer.gov/data/options.html). Requests for data access can be sent by following the instructions on that website. Permission to access the research data files was obtained with the reference number 11375-Nov2014. The study contained no personal identifying information and required no informed consent. Anonymous patient’s data were extracted from online SEER registries, which require no further institutional review approval prior to use. The study was approved by the Review Board of Nanjing Medical University, Nanjing, China.

### Statistical Analysis

The relation of age (younger vs older) with clinicopathological features was analyzed by chi-square (χ2) test. Comparisons of continuous variables between two groups were completed using Student’s *t*-test. Survival was analyzed based on age (younger vs older) and was stratified by AJCC stages I–IV, or unknown stage. Survival curves were provided by Kaplan–Meier estimates; differences between two groups were analyzed by log-rank test. Multivariate Cox regression models were performed to evaluate risk factors for survival outcomes. All statistical analyses were completed using the statistical software Statistical Package for Social Sciences (SPSS) for Windows, version 17 (SPSS Inc., Chicago, IL, USA). *P* < 0.05 was considered significant.

## Results

### Clinicopathological differences between younger and older groups

We identified 123,356 eligible patients with CC in the SEER database during the 10-year study period (1996–2005), of whom 63,436 (51.4%) were males and 59,920 (48.6%) were females; and 4,504 (3.6%) were no older than 40 years (younger patients), and 118,852 (96.4%) were older than 40 years (older patients). The median follow up time was 69 months (interquartile range [IQR]: 22–106 months) in the younger group and 65 months (IQR: 18–100 months) in the older group.

Among younger patients, CC was more frequent during 2003–2005 (*P*<0.05) and less frequent among Caucasians (*P*<0.001) than for older patients. Younger patients also had significantly more poorly or undifferentiated tumors (*P*<0.001), more mucinous or signet-ring histologies (*P*<0.001), presented with more advanced AJCC stages (III/IV, *P*<0.001), and underwent more surgery (*P*<0.001). The younger and older groups did not significantly differ by sex (*P* = 0.14; Table 1).

**Table 1:** Characteristics of patients from the SEER database by age.

	Total	Younger Group	Older Group	P value
Characteristic	(n = 123,356)	(n = 4504)	(n = 118,852)	
Median follow up (months)	65	69	65	P<0.001
(IQR)	18–100	22–106	18–100	
Years of diagnosis				0.027
1996–2002	76270	2714	73556	
2003–2005	47086	1790	45296	
Sex				0.138
male	63436	2365	61071	
female	59920	2139	57781	
Race				P<0.001
Caucasian	97758	3292	94466	
African-American	14943	674	14269	
Others*	10655	538	10117	
Pathological grading				P<0.001
High/ Moderate	88209	2940	85269	
Poor/ undifferentiated	23607	1136	22471	
Unknown	11540	428	11112	
Histological Type				P<0.001
Adenocarcinoma	107438	3573	103865	
Mucinous/Signet-ring cancer	15918	931	14987	
AJCC stage				P<0.001
I	19996	429	19567	
II	31946	1072	30874	
III	32064	1433	30631	
IV	26512	1199	25313	
Unknown	12838	371	12467	
Surgery performed				P<0.001
Yes	113303	4188	109115	
No	9578	290	9288	
Unknown	475	26	449	

*Including American Indian/AK Native, Asian/Pacific Islander, and unknowns.

### Impact of age on survival outcomes in patients with CC treated with surgery

**Fig 1:**
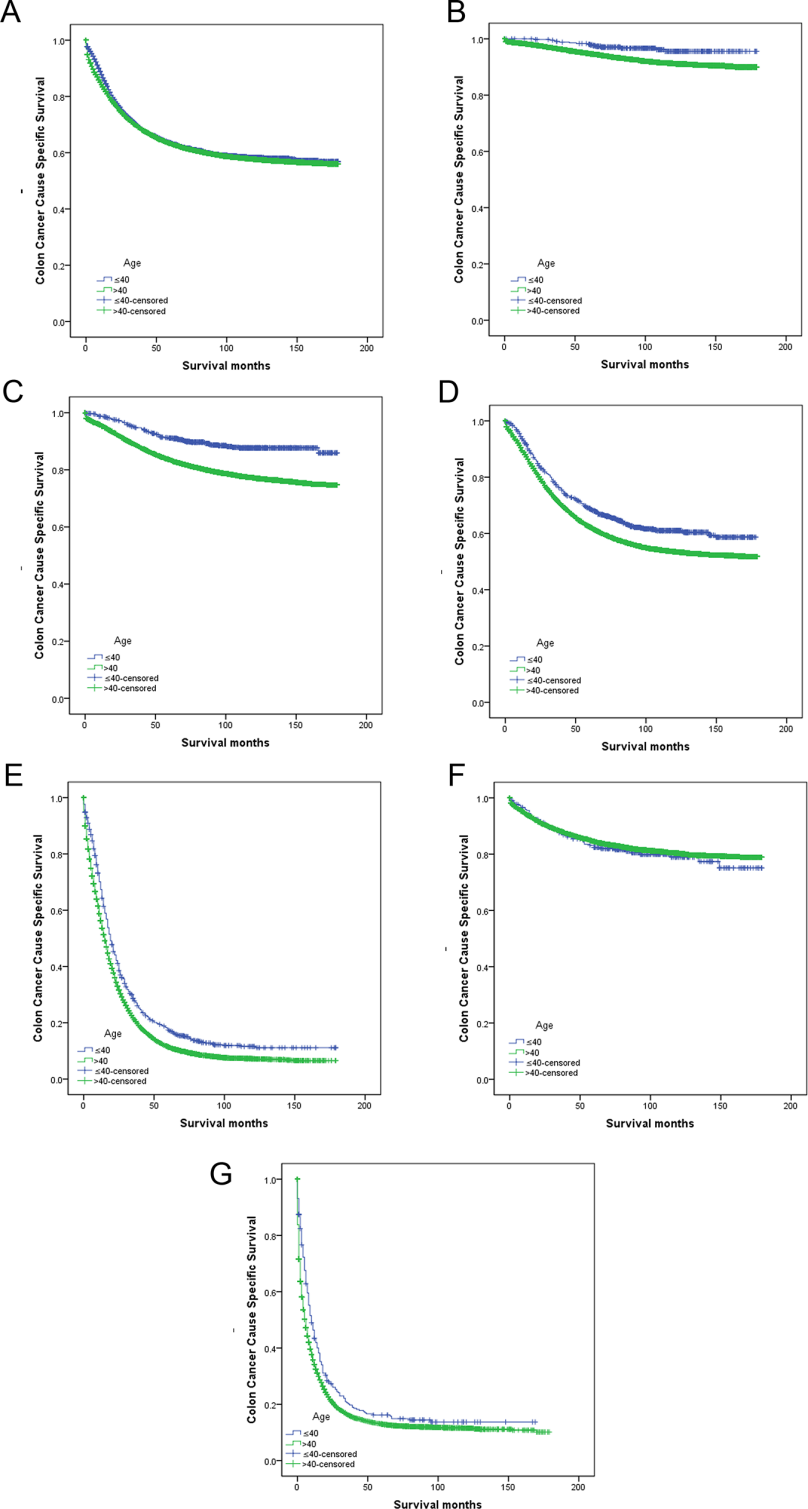
Survival curves for colon cancer patients at younger (≤ 40 years) and older (> 40 years) group. (A) All patients treated with surgery, χ^2^ = 0.033, *P* = 0.86. (B) Stage I colon cancer treated with surgery, χ^2^ = 10.368, *P*<0.001. (C) Stage II colon cancer treated with surgery, χ^2^ = 56.935, *P*<0.001; (D) Stage III colon cancer treated with surgery, χ2 = 27.752, *P*<0.001. (E) Stage IV colon cancer treated with surgery, χ^2^ = 45.775, *P*<0.001. (F) Unknown stage patients treated with surgery, χ2 = 0.410, *P* = 0.52. (G) Patients with colon cancer treated without surgery, χ^2^ = 12.760, *P*<0.001.

Overall 5-year CCSS did not significantly differ for the two age groups (younger: 63.4%; older: 63.2%; χ^2^ = 2.089, *P* = 0.15, univariate log-rank test), nor did it significantly differ among all patients treated with surgery (younger: 66.7%; older: 67.3%; χ^2^ = 0.03; *P* = 0.86; Table 2, Fig 1A). However, when 5-year CCSS was further stratified by disease stage, younger patients who had undergone surgery had significantly better survival than older, surgically treated patients at the same disease stage for stages I–IV (Table 2; Fig 1B–1E). Counterintuitively, among surgically treated patients with unknown disease stages, older patients had a higher 5-year CCSS rate (84.5%) than did younger patients (82.4%) but not significantly so (*P* = 0.52; Table 2, Fig 1F). In multivariate analysis (Cox regression) age was an independent survival factor in stages I–IV (Table 3).

**Table 2:** Univariate analysis of age on colon cancer-specific survival by disease stage.

Variable	*n*	5-year survival (%)	Log rank χ^2^ test	P
Surgery performed				
Age			0.033	0.855
≤40	4188	66.7%		
41–80	109115	67.3%		
Stage I				
Age			10.368	P = 0.001
≤40	427	98.1%		
41–80	19523	94.7%		
Stage II				
Age			56.935	P<0.001
≤40	1068	91.1%		
41–80	30788	83.5%		
Stage III				
Age			27.752	P<0.001
≤40	1427	68.8%		
41–80	30550	62.3%		
Stage IV				
Age			45.775	P<0.001
≤40	972	17.4%		
41–80	18802	11.4%		
Unknown				
Age			0.410	P = 0.522
≤40	294	82.4%		
41–80	9452	84.5%		
No Surgery performed				
Age			12.760	P<0.001
≤40	290	16.2%		
41–80	9288	12.9%		

**Table 3:** Multivariate Cox model analyses of prognostic factors for colon cancer at different stages.

Variable	Hazard Ratio	95%CI	*P*
Surgery performed			
Stage I			
Age			0.001*
≤40	1.000	Reference	
41–80	2.319	1.394–3.858	
Stage II			
Age			
≤40	1.000	Reference	P<0.001*
41–80	1.856	1.544–2.232	
Stage III			
Age			
≤40	1.000	Reference	P<0.001*
41–80	1.309	1.198–1.430	
Stage IV			
Age			
≤40	1.000	Reference	P<0.001*
41–80	1.288	1.200–1.382	
No Surgery performed			
Age			P<0.001**
≤40	1.000	Reference	
41–80	1.281	1.124–1.459	

*P* values refer to comparison between two groups.

*Adjusted for years of diagnosis, sex, age, race, pathological grading, tumor histology and number of dissected lymph nodes, as covariates

** Adjusted for years of diagnosis, sex, age, race, pathological grading, tumor histology and AJCC stage as covariates.

### Impact of age on survival outcomes in non-surgically treated patients

In patients not treated with surgery, 5-year CCSS significantly differed by age group (younger: 16.2%; older: 12.9%; χ^2^ = 12.760, *P*<0.001; Table 2; Fig 1G). In multivariable analysis, older patients were 1.281 times more likely to die of colon cancer than were younger patients (Table 3).

## Discussion

A sharp increase over recent decades in the number of younger patients diagnosed with CC has been reported in several countries [5–7]. Its reported incidence among patients of 20–40 years of age increased by 17% during 1973–1999 [21]. We found that incidence of CC increased more quickly during 2003–2005 than during 1996–2003 (*P* = 0.03), which indicated a need for the increased incidence in this population to be further investigated with an eye to prevention and early detection. Previous studies are somewhat complicated by the lack of a standard definition of young CC patients [2, 13, 18, 22–25]. We used the cutoff age of 40 years, in line with most denoted studies, though upper limits have ranged from 30 to 50 years [26–28].

The prognosis of CC in younger patients is controversial. Various studies have reported poorer prognosis among young patients with CC than in older patients. This has been attributed to more advanced disease at diagnosis, tumor stage [13, 14, 29], more mucinous or signet-ring histopathology and poorly differentiated tumors [12, 15]. These patterns were also seen in our study. Although the younger patient group had a significantly higher incidence of inferior prognostic factors, they had better stage-specific 5-year CCSS the older-onset cohort, as shown in both univariate and multivariate analysis. Our results were similar to those of other studies [16–19]. In this study, we analyzed 4,504 younger CC patients over a 10-year time period, using a much larger cohort than any other comparable study. We also excluded patients over age of 80 years for their short life expectation.

In general, surgeons are more inclined to use all therapeutic options in young patients as they are in better health and are more able to tolerate toxicities associated with chemotherapy and suffer from fewer postoperative complications [9, 30–34]. Although studies of equal-access cohorts have shown younger patients with CC present with more advanced disease and higher rates of disease recurrence, their overall survival exceeds that of older cohorts, which may be partly due to their greater use of adjuvant therapy and more palliative multidisciplinary postoperative treatment [9, 17]. Poor tolerance to treatment because of poor performance status or the presence of other comorbidities may contribute to inferior survival of older patients. Our data also showed that older patients undergo a lower rate of surgery than younger patients, even if their clinicopathological characteristics are relatively good. Patients with stage IV CRC managed with primary tumor resection were shown to have significantly better survival than to those managed with chemotherapy alone [35, 36]. Less aggressive treatments offered to patients with limited comorbidities are likely to affect their outcomes [37]. Younger patients also have a higher proportion of tumors that show microsatellite instability, which may contribute to their better survival. Studies found that patients with MSI tumors are apparently less likely to metastasize lymph nodes and distal organs, regardless of depth of tumor invasion, compared with microsatellite-stable tumors [38–40].

Younger patients who did not undergo surgery still had better 5-year CCSS than older ones. Our analysis of the SEER data, which are nationally representative and use a large, unselected group of patients with no surgical therapy, showed that younger patients had better 5-year CCSS than older ones. Although a clinical trial showed that survival of patients with advanced CRC was significantly increased when incorporating new therapeutic agents [41], a meta-analysis of randomized clinical trials of newer chemotherapy agents between 1995 and 2004, which compared younger and older patients with advanced CRC, showed similar survival in both groups[42]. Patients enrolled in clinical trials are always strictly selected and under intense supervision, which may explain their inconsistent results from ours. This study adds to current knowledge by answering more in-depth research questions about age and prognosis through analysis of population-based data from the large SEER database. However, it has several potential limitations. First, the SEER database lacks important information on tumor characteristics and specific cancer therapies, such as perineural invasion, lymphovascular invasion, neoadjuvant or adjuvant therapy, quality of surgery, etc. Thus, our study could not adjust for these potential confounding factors. Second, SEER data do not separate surgeries with palliative intent from those with curative intent. Thus, the beneficial effect of surgery on survival may be underestimated, specifically those received radical resection. Finally, the SEER database does not include information on comorbidities, which limits our ability to calculate their effect on CCSS. Despite these potential limitations, there are many merits to this population-based study. First, we can analyze 123,356 patients with colon cancer during a 10-year time period, much larger than any other homologous study. Second, the SEER database is nationally representative, because its registries cover 28% of the United States population, which is ethnically and socioeconomically diverse. Consequently, these finding may be generalizable to the entire United States.

In summary, patients with CC who are aged 40 and younger have higher CCSS rates than their older counterparts, both after surgery by stage, and among those who are not treated with surgery. Clinicians should consider age in their assessments and treatment decisions for patients with CC.

## Abbreviations

SEER: Surveillance, Epidemiology and End Results
CC: colon cancer
AJCC: American Joint Committee on Cancer, CSS(Cancer-Specific Survival)

## References

[1] Siegel R, Desantis C, Jemal A. Colorectal cancer statistics, 2014. CA Cancer J Clin. 2014;64(2):104–17. 10.3322/caac.21220 . 24639052

[2] Taggarshe D, Rehil N, Sharma S, Flynn JC, Damadi A. Colorectal cancer: are the "young" being overlooked? American journal of surgery. 2013;205(3):312–6; discussion 6. Epub 2013/02/19. 10.1016/j.amjsurg.2012.10.016 . 23414955

[3] Atkin WS, Cuzick J, Northover JM, Whynes DK. Prevention of colorectal cancer by once-only sigmoidoscopy. Lancet. 1993;341(8847):736–40. Epub 1993/03/20. . 8095636 10.1016/0140-6736(93)90499-7

[4] Eheman C, Henley SJ, Ballard-Barbash R, Jacobs EJ, Schymura MJ, Noone AM, et al Annual Report to the Nation on the status of cancer, 1975–2008, featuring cancers associated with excess weight and lack of sufficient physical activity. Cancer. 2012;118(9):2338–66. Epub 2012/03/31. 10.1002/cncr.27514 . 22460733 PMC4586174

[5] Edwards BK, Ward E, Kohler BA, Eheman C, Zauber AG, Anderson RN, et al Annual report to the nation on the status of cancer, 1975–2006, featuring colorectal cancer trends and impact of interventions (risk factors, screening, and treatment) to reduce future rates. Cancer. 2010;116(3):544–73. Epub 2009/12/10. 10.1002/cncr.24760 19998273 PMC3619726

[6] You YN, Xing Y, Feig BW, Chang GJ, Cormier JN. Young-onset colorectal cancer: is it time to pay attention? Archives of internal medicine. 2012;172(3):287–9. Epub 2011/12/14. 10.1001/archinternmed.2011.602 . 22157065

[7] Atrkar-Roushan Z, Kazemnejad A, Mansour-Ghanaei F, Zayeri F. Trend analysis of gastrointestinal cancer incidences in Guilan province: comparing rates over 15 years. Asian Pacific journal of cancer prevention: APJCP. 2013;14(12):7587–93. Epub 2014/01/28. . 24460338 10.7314/apjcp.2013.14.12.7587

[8] Li J, Wang Z, Yuan X, Xu L, Tong J. The prognostic significance of age in operated and non-operated colorectal cancer. BMC cancer. 2015;15:83 Epub 2015/04/18. 10.1186/s12885-015-1071-x 25885448 PMC4345025

[9] Schellerer VS, Merkel S, Schumann SC, Schlabrakowski A, Fortsch T, Schildberg C, et al Despite aggressive histopathology survival is not impaired in young patients with colorectal cancer: CRC in patients under 50 years of age. International journal of colorectal disease. 2012;27(1):71–9. Epub 2011/09/02. 10.1007/s00384-011-1291-8 . 21881876

[10] Makela JT, Kiviniemi H. Clinicopathological features of colorectal cancer in patients under 40 years of age. International journal of colorectal disease. 2010;25(7):823–8. Epub 2010/03/11. 10.1007/s00384-010-0914-9 . 20217423

[11] Jones HG, Radwan R, Davies M, Evans M, Khot U, Chandrasekaran TV, et al Clinicopathological characteristics of colorectal cancer presenting under the age of 50. International journal of colorectal disease. 2015;30(4):483–9. Epub 2015/02/25. 10.1007/s00384-015-2166-1 . 25707594

[12] Plunkett M, Murray M, Frizelle F, Teague L, Hinder V, Findlay M. Colorectal adenocarcinoma cancer in New Zealand in those under 25 years of age (1997–2007). ANZ journal of surgery. 2014;84(5):371–5. Epub 2013/10/10. 10.1111/ans.12380 . 24102993

[13] Fu J, Yang J, Tan Y, Jiang M, Wen F, Huang Y, et al Young patients (</ = 35 years old) with colorectal cancer have worse outcomes due to more advanced disease: a 30-year retrospective review. Medicine. 2014;93(23):e135 Epub 2014/11/22. 10.1097/md.0000000000000135 . 25415667 PMC4616343

[14] Chan KK, Dassanayake B, Deen R, Wickramarachchi RE, Kumarage SK, Samita S, et al Young patients with colorectal cancer have poor survival in the first twenty months after operation and predictable survival in the medium and long-term: analysis of survival and prognostic markers. World journal of surgical oncology. 2010;8:82 Epub 2010/09/16. 10.1186/1477-7819-8-82 20840793 PMC2954852

[15] Keswani SG, Boyle MJ, Maxwell JPt, Mains L, Wilks SM, Hunt JP, et al Colorectal cancer in patients younger than 40 years of age. The American surgeon. 2002;68(10):871–6. Epub 2002/11/05. . 12412713

[16] Zahir MN, Azhar EM, Rafiq S, Ghias K, Shabbir-Moosajee M. Clinical features and outcome of sporadic colorectal carcinoma in young patients: a cross-sectional analysis from a developing country. ISRN oncology. 2014;2014:461570 Epub 2014/07/10. 10.1155/2014/461570 25006505 PMC4004039

[17] Steele SR, Park GE, Johnson EK, Martin MJ, Stojadinovic A, Maykel JA, et al The impact of age on colorectal cancer incidence, treatment, and outcomes in an equal-access health care system. Dis Colon Rectum. 2014;57(3):303–10. Epub 2014/02/11. 10.1097/DCR.0b013e3182a586e7 00003453-201403000-00004 [pii]. . 24509451

[18] Yeo SA, Chew MH, Koh PK, Tang CL. Young colorectal carcinoma patients do not have a poorer prognosis: a comparative review of 2,426 cases. Techniques in coloproctology. 2013;17(6):653–61. Epub 2013/03/06. 10.1007/s10151-013-0977-z . 23460362

[19] Austin H, Henley SJ, King J, Richardson LC, Eheman C. Changes in colorectal cancer incidence rates in young and older adults in the United States: what does it tell us about screening. Cancer causes & control: CCC. 2014;25(2):191–201. Epub 2013/11/20. 10.1007/s10552-013-0321-y 24249437 PMC4394895

[20] Warren JL, Klabunde CN, Schrag D, Bach PB, Riley GF. Overview of the SEER-Medicare data: content, research applications, and generalizability to the United States elderly population. Med Care. 2002;40(8 Suppl):IV-3-18. Epub 2002/08/21. 10.1097/01.mlr.0000020942.47004.03 . 12187163

[21] O'Connell JB, Maggard MA, Liu JH, Etzioni DA, Livingston EH, Ko CY. Rates of colon and rectal cancers are increasing in young adults. The American surgeon. 2003;69(10):866–72. Epub 2003/10/23. . 14570365

[22] Mitry E, Benhamiche AM, Jouve JL, Clinard F, Finn-Faivre C, Faivre J. Colorectal adenocarcinoma in patients under 45 years of age: comparison with older patients in a well-defined French population. Diseases of the colon and rectum. 2001;44(3):380–7. Epub 2001/04/06. . 11289284 10.1007/BF02234737

[23] Bouassida M, Feidi B, Mroua B, Chtourou MF, Sassi S, Chebbi F, et al Histopathologic characteristics and short-term outcomes of colorectal cancer in young Tunisian patients: one center's experience. The Pan African medical journal. 2012;12:10 Epub 2012/07/25. 22826734 PMC3396856

[24] Benmoussa A, Zamiati S, Badre W, Wakadi A, Bennani N, Tahiri Jouti N, et al Colorectal cancer: comparison of clinicopathologic features between Moroccans patients less than 50 years old and older. Pathologie-biologie. 2013;61(3):117–9. Epub 2012/03/01. 10.1016/j.patbio.2012.01.003 . 22361163

[25] Neufeld D, Shpitz B, Bugaev N, Grankin M, Bernheim J, Klein E, et al Young-age onset of colorectal cancer in Israel. Techniques in coloproctology. 2009;13(3):201–4. Epub 2009/07/18. 10.1007/s10151-009-0501-7 . 19609485

[26] Dozois EJ, Boardman LA, Suwanthanma W, Limburg PJ, Cima RR, Bakken JL, et al Young-onset colorectal cancer in patients with no known genetic predisposition: can we increase early recognition and improve outcome? Medicine. 2008;87(5):259–63. Epub 2008/09/17. 10.1097/MD.0b013e3181881354 18794708 PMC4437192

[27] Li M, Li JY, Zhao AL, Gu J. Do young patients with colorectal cancer have a poorer prognosis than old patients? The Journal of surgical research. 2011;167(2):231–6. Epub 2011/02/15. 10.1016/j.jss.2010.10.040 . 21316708

[28] Ganapathi S, Kumar D, Katsoulas N, Melville D, Hodgson S, Finlayson C, et al Colorectal cancer in the young: trends, characteristics and outcome. International journal of colorectal disease. 2011;26(7):927–34. Epub 2011/03/23. 10.1007/s00384-011-1174-z . 21424713

[29] Ben-Ishay O, Brauner E, Peled Z, Othman A, Person B, Kluger Y. Diagnosis of colon cancer differs in younger versus older patients despite similar complaints. The Israel Medical Association journal: IMAJ. 2013;15(6):284–7. Epub 2013/07/26. . 23882892

[30] Ohtani H, Arimoto Y, Nishio K, Kanamiya Y, Oba H, Adachi K, et al Efficacy and toxicity of fluorouracil, leucovorin plus oxaliplatin (FOLFOX4 and modified FOLFOX6) followed by fluorouracil, leucovorin plus irinotecan(FOLFIRI)for advanced or metastatic colorectal cancer—case studies. Gan to kagaku ryoho Cancer & chemotherapy. 2008;35(10):1769–74. Epub 2008/10/22. . 18931586

[31] Kang BW, Kim TW, Lee JL, Ryu MH, Chang HM, Yu CS, et al Bevacizumab plus FOLFIRI or FOLFOX as third-line or later treatment in patients with metastatic colorectal cancer after failure of 5-fluorouracil, irinotecan, and oxaliplatin: a retrospective analysis. Medical oncology (Northwood, London, England). 2009;26(1):32–7. Epub 2008/05/24. 10.1007/s12032-008-9077-8 . 18498064

[32] Goodwin RA, Asmis TR. Overview of systemic therapy for colorectal cancer. Clin Colon Rectal Surg. 2009;22(4):251–6. Epub 2010/11/03. 10.1055/s-0029-1242465 21037816 PMC2796098

[33] Chew MH, Koh PK, Ng KH, Eu KW. Improved survival in an Asian cohort of young colorectal cancer patients: an analysis of 523 patients from a single institution. International journal of colorectal disease. 2009;24(9):1075–83. Epub 2009/04/24. 10.1007/s00384-009-0701-7 . 19387661

[34] Stillwell AP, Buettner PG, Ho YH. Meta-analysis of survival of patients with stage IV colorectal cancer managed with surgical resection versus chemotherapy alone. World J Surg. 2010;34(4):797–807. Epub 2010/01/08. 10.1007/s00268-009-0366-y . 20054541

[35] Yang Z, Chen H, Liao Y, Xiang J, Kang L, Wang L, et al Clinicopathological characteristics and long-term outcomes of colorectal cancer in elderly Chinese patients undergoing potentially curative surgery. Surgery today. 2014;44(1):115–22. Epub 2013/02/27. 10.1007/s00595-013-0507-7 . 23440360

[36] Nitsche U, Spath C, Muller TC, Maak M, Janssen KP, Wilhelm D, et al Colorectal cancer surgery remains effective with rising patient age. International journal of colorectal disease. 2014;29(8):971–9. Epub 2014/06/14. 10.1007/s00384-014-1914-y 24924447 PMC4101253

[37] Lemmens VE, Janssen-Heijnen ML, Verheij CD, Houterman S, Repelaer van Driel OJ, Coebergh JW. Co-morbidity leads to altered treatment and worse survival of elderly patients with colorectal cancer. The British journal of surgery. 2005;92(5):615–23. Epub 2005/03/22. 10.1002/bjs.4913 . 15779071

[38] Kirzin S, Marisa L, Guimbaud R, De Reynies A, Legrain M, Laurent-Puig P, et al Sporadic early-onset colorectal cancer is a specific sub-type of cancer: a morphological, molecular and genetics study. PloS one. 2014;9(8):e103159 Epub 2014/08/02. 10.1371/journal.pone.0103159 25083765 PMC4118858

[39] Laskar RS, Talukdar FR, Mondal R, Kannan R, Ghosh SK. High frequency of young age rectal cancer in a tertiary care centre of southern Assam, North East India. The Indian journal of medical research. 2014;139(2):314–8. Epub 2014/04/11. 24718409 PMC4001346

[40] Hubbard JM, Grothey A. Adolescent and young adult colorectal cancer. Journal of the National Comprehensive Cancer Network: JNCCN. 2013;11(10):1219–25. Epub 2013/10/22. . 24142823 10.6004/jnccn.2013.0144

[41] Grothey A, Goldberg RM. A review of oxaliplatin and its clinical use in colorectal cancer. Expert Opin Pharmacother. 2004;5(10):2159–70. Epub 2004/10/06. 10.1517/14656566.5.10.2159 . 15461551

[42] Blanke CD, Bot BM, Thomas DM, Bleyer A, Kohne CH, Seymour MT, et al Impact of young age on treatment efficacy and safety in advanced colorectal cancer: a pooled analysis of patients from nine first-line phase III chemotherapy trials. Journal of clinical oncology: official journal of the American Society of Clinical Oncology. 2011;29(20):2781–6. Epub 2011/06/08. 10.1200/jco.2010.33.5281 . 21646604 PMC4874194

